# Microfluidics applications for high-throughput single cell sequencing

**DOI:** 10.1186/s12951-021-01045-6

**Published:** 2021-10-11

**Authors:** Wen-min Zhou, Yan-yan Yan, Qiao-ru Guo, Hong Ji, Hui Wang, Tian-tian Xu, Bolat Makabel, Christian Pilarsky, Gen He, Xi-yong Yu, Jian-ye Zhang

**Affiliations:** 1 Key Laboratory of Molecular Target & Clinical Pharmacology , The State & NMPA Key Laboratory of Respiratory Disease, School of Pharmaceutical Sciences & the Fifth Affiliated Hospital, Guangzhou Medical University, Guangzhou, 511436 People’s Republic of China; 2 School of Medicine, Shanxi Datong University, Datong, 037009 People’s Republic of China; 3 Guangzhou Institute of Pediatrics/Guangzhou Women and Children’s Medical Center, Guangzhou Medical University, Guangzhou, 510623 People’s Republic of China; 4 Xinjiang Institute of Materia Medica, Urumqi, 830004 People’s Republic of China; 5 Department of Surgery, Friedrich-Alexander University of Erlangen-Nuremberg (FAU), University Hospital of Erlangen, Erlangen, Germany

**Keywords:** Single cell separation, Single cell RNA sequencing (scRNA-seq), High-throughput, Microfluidic, Droplets, Biomedical applications

## Abstract

The inherent heterogeneity of individual cells in cell populations plays significant roles in disease development and progression, which is critical for disease diagnosis and treatment. Substantial evidences show that the majority of traditional gene profiling methods mask the difference of individual cells. Single cell sequencing can provide data to characterize the inherent heterogeneity of individual cells, and reveal complex and rare cell populations. Different microfluidic technologies have emerged for single cell researches and become the frontiers and hot topics over the past decade. In this review article, we introduce the processes of single cell sequencing, and review the principles of microfluidics for single cell analysis. Also, we discuss the common high-throughput single cell sequencing technologies along with their advantages and disadvantages. Lastly, microfluidics applications in single cell sequencing technology for the diagnosis of cancers and immune system diseases are briefly illustrated.

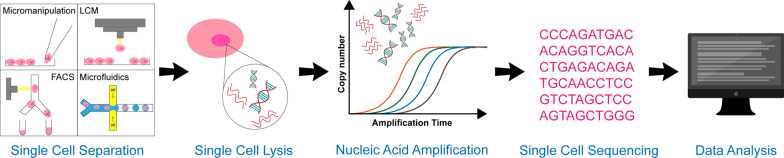

## Introduction

A single cell is the basic structural and functional unit of living organisms. Cells derived from the same type of cells under the same external stimulus or physiological conditions may exhibit cell-to-cell differences [1]. Numerous traditional studies, such as cell differentiation and gene expression, focused on a cell population, which is the “whole” characterization of multiple cells. However, it was confirmed that cellular heterogeneity and multi-modal distributions commonly existed in an isogenic or clonal population. Therefore, the outcome of studying a population of cells might not be accurate. In other words, heterogeneity can be found in the morphology, composition, functions and genetic behaviors of cells that look same. The analysis of heterogeneous cell populations in bulk is only able to provide averaged data about the multiple cells [2–4]. Due to the cellular heterogeneity, the genetic information of the same phenotype of cells may be significantly different, and many low abundance information may be lost in the background [5]. To solve this dilemma, it is needed to analyze a population of cells at the level of a single cell. Therefore, single cell analysis has become an attractive and challenging field of modern biomedical research.

**Fig. 1:**
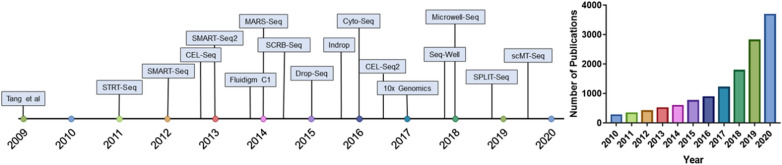
Timeline of single cell sequencing milestones and the publications of single cell sequencing in the past ten years (2010–2020). Literature search was performed using Web of Science to determine the number of publications on single cell sequencing

With the rapid development of next generation sequencing (NGS) technology, more and more sequencing technology platforms have emerged and become the foundation of precision medicine. To explore the variability between individual cells and to compensate for the limitations of traditional sequencing technology, development of single cell sequencing technology is terribly demanded [6]. In 2009, Tang’s group firstly reported the mRNA-seq whole-transcriptome analysis method on a mammalian single cell [7]. In 2011, Navin et al. firstly achieved the genome sequencing of a single human cell to investigate the structure and evolution of the tumor cell population in breast cancer [8]. Since being rated as the Method of the Year for 2013, single cell sequencing for genetic material has become a routine in investigating cell-to-cell heterogeneity [9]. The Journal of Science indicated that single cell sequencing tackled basic and biomedical questions and introduced microfluidics device that isolated single cells and copied their DNA [10]. Single cell sequencing has been applied to analyze genetic information and identify cell subtypes, and it provided valuable insights for disease diagnosis and treatment [11]. The timeline of single cell sequencing milestones and the publications of single cell sequencing in the past 10 years (2010–2020) are shown in Fig. 1. Additionally, the rapid developments of these technologies are discussed in details hereinbelow.

In the early 1990s, Manz and Widmer proposed a concept of miniaturized total chemical analysis system (μTAS), which made analysis of biological samples more efficient and sensitive [12]. Microfluidics, the key component of μTAS, has been rapidly developed into a leading-edge research method in the field of life sciences and analytical chemistry. Microfluidics, also known as “lab-on-a-chip”, is a novel tool to analyze single cells in an easy and efficient manner [13]. Microfluidics provides several benefits over conventional techniques in analyzing samples. Firstly, microfluidic chip is flexible in designing structures and functions to meet the demands of single cell analysis [14]. Secondly, typical microfluidic channels have dimension of tens to hundreds of microns that work from picoliter to nanoliter volumes of solution, enabling reduction of sample loss and high sensitivity, and making high-throughput single cell analysis possible [15]. In addition, the integration of multifunctional units and microfluidic chips can achieve automation, preventing measurement errors generated from human operations [16]. By far, single cell analysis based on multi-technical combinational microfluidics has revolutionized plenty of research fields, and its application on single cell sequencing has received broad attention for its high-throughput characteristics.

In this manuscript, we first introduce the processes of single cell sequencing including single cell separation, single cell lysis, nucleic acid amplification, high-throughput sequencing, data processing and data analysis. We then summarize the principles of microfluidics for single cell analysis, such as traps-based, vavles-based, and droplet-based methods. Also, we discuss the advantages and disadvantages of common high-throughput single cell RNA sequencing (scRNA-seq) technologies. Lastly, microfluidics applications of single cell sequencing technology in the diagnosis of cancers and immune system diseases are briefly illustrated as well.

## Process of single cell sequencing

**Fig. 2:**
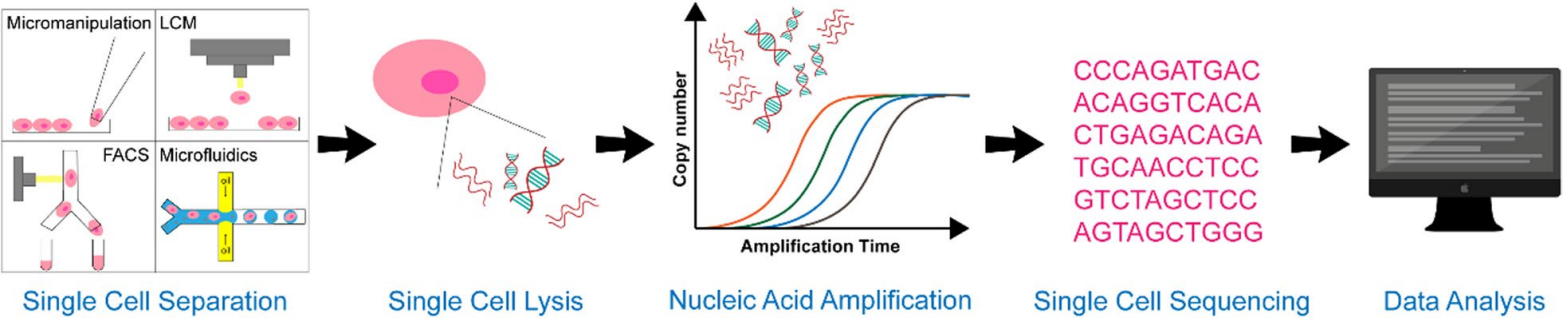
Processes of single cell sequencing. The main processes of single cell sequencing include single cell separation (such as micromanipulation, LCM, FACS and microfluidics), single cell lysis, nucleic acid amplification, high-throughput sequencing, data processing, and data analysis

Single cell sequencing is an emerging technology that separates cell population in tissues or body fluids into a single cell and performs high-throughput sequencing analysis of its genetic material [17]. The main process of single cell sequencing includes single cell separation, single cell lysis, nucleic acid amplification, high-throughput sequencing, data processing and data analysis (Fig. 2) [18].

### Single cell separation

Different from traditional sequencing methods, the separation of single cells is the key to this technology, which must be fast, effective, and gentle to maintain the native expression profiles. The first step in single cell analysis is to isolate individual cell samples for examination. At present, the approaches of separating single cell samples include limiting dilution, micromanipulation, laser capture microdissection (LCM), fluorescence activated cell sorting (FACS) and microfluidic technology. Traditional separation methods have already been reviewed in detail elsewhere [19–22] and are only briefly discussed. In this section, we focus on microfluidic methods and a detailed introduction is presented in Section “Principles of microfluidics for single cell analysis”.

These common single cell separation methods are well-established and standardized techniques with a wide range of applications [23, 24]. Limiting dilution mainly uses hand-pipettes or pipetting robots to isolate individual cells, whose probability for the number of cells per aliquot obey Poisson’s distribution. This method is obsolete because it cannot exclude some small cell populations [25]. Micromanipulation for manual cell picking works through combining microscope and micropipette, and applying suction to capture individual cells. Cells obtained from micromanipulation might suffer from mechanical injury [26]. LCM is an advanced tool to quickly and precisely acquire single cells or cell compartments from mostly solid tissue samples after slice and dyeing treatment. This method successfully maintains cell morphology and structure and preserves the spatial location information [27]. FACS systems provide the ability to sort individual cells via labeling with fluorescent tags on the specific surface molecule of cells in various types of flow cytometers. The cell stream rapidly passes through a laser beam to provide optical excitation and then optical detectors in downstream are used to capture cells with specific signals [28].

Microfluidics is novel technology which precisely controls fluid in the micro-scale, with the characteristics of small size, low sample consumption, fast reaction speed, high sorting accuracy and flexible operation [29]. A great deal of microfluidic devices have been proposed for single cell analysis in the past years [30] and we focus on the ability of the device on isolation of individual cells for further downstream analysis and culture. The microfluidic device has a multi-channel structure, and channels ranging from 10 to 100 μm wide enable them to fit the size and volume of each single cells. Besides, the channel diameter and fluid velocity can be flexibly modified and adjusted to design a variety of functions [31]. Research showed that most of the microfluidic devices use the following microfluidic principles to isolate individual cells: hydrodynamic cell traps, pneumatic membrane valves, and droplet-in-oil-based isolation [32]. More importantly, the most popular microfluidic isolation methods currently are applying microdroplets to encapsulate single cells in an inert carrier oil, which creates an enclosed space that reduces the risk of sample contamination [33].

Collectively, the outcomes and characteristics of above methods are quite different. Limiting dilution, micromanipulation and LCM are time-consuming and laborious, allowing only limited throughput [34]. FACS is high-throughput and is extremely efficient in cell sorting. Microfluidics empowers performing compartmentalization of up to thousands of cells in a very short time, which is an ideal technique for large-scale applications [35]. The merits and limitations of the above-mentioned methods are compared and summarized in Table 1.

**Table 1:** Comparison of different single cell isolation methods

Methods	Description	Isolation process	Applicability	Throughput (cells per run)	Cost	Merits	Limitations
Limiting dilution	Application of hand pipettes or pipetting robots to isolate single cells through dilution of the cell suspension	Manual/semi-automatic	Suspension cells	Low (< 100)	Low	Simple operation	Low specificity
							Low efficiency
							Low precision
							Cell loss
							Low work capacity (< 100)
Micromanipulation	Application of inverted microscope combined with micropipettes to select and isolate single cells	Manual	Suspension cells	Low (< 100)	Low	Simple operation	Low efficiency
						Flexible sampling	Mechanical injury
						Visualized operation	High difficulty
							Low work capacity (< 100)
LCM	Application of infrared laser under a microscope to isolate single cell or cell compartments from solid tissue samples	Manual	Tissue samples	Low (< 100)	High	Maintain integrity of sample	Nuclear damage
							Genetic material loss
							RNA pollution
							High difficulty
							Low work capacity (< 100)
FACS	Application of fluorescence labeling specific molecules on the cell surface to sort cells	Semi-automatic	Suspension cells	High (> 1000)	High	·High specificity	Mechanical injury
						·High accuracy	Large sample amount
						·High sensitivity	Cannot process cells less than 1000
Traps-based microfluidics	Application of microfluidic chips to separate single cells through traps	Semi-automatic	Suspension cells	High (> 1000)	High	Flexible operation	Low specificity
						Efficient cell pairing and fusion	Partial stimulation on cells
Valves-based microfluidics	Application of microfluidic chips to separate single cells through valves	Semi-automatic	Suspension cells	High (> 1000)	High	High sensitivity	Difficult and time-consuming fabrication
						High automation	Not portable
						Low sample volume	
Droplet-based microfluidics	Application of microfluidic chips to separate single cells through droplets	Semi-automatic	Suspension cells	High (1000–10,000)	High	High sensitivity	Random encapsulation
						High specificity	Complex equipment
						Noise-free	

*LCM* Laser capture microdissection, *FACS* fluorescence activated cell sorters

### Single cell lysis

Due to the small quantities and volumes of analytes involved in single cell analysis, manipulation strategies must be carefully chosen [36]. Single cell lysis plays a significant role in the multi-omics analysis. Compared with traditional methods, microfluidics-based lysis minimizes lysate dilution, thus increasing assay sensitivity to a large extent [37]. Here, we briefly discussed common cell lysis methods, such as physical, chemical and enzymatic methods.

There are three main forms of physical cell lysis: mechanical, thermal and electrical. Mechanical lysis separates cell membranes mainly through a mechanical force; thermal lysis depends on heat-induced denaturation of cell membrane proteins and requires additional temperature cycling; and electrical lysis destroys the cell membranes via the electric field-induced molecular reorientation [38]. Wei et al. established a new low-voltage controllable method for cell lysis on a microfluidic chip, and a rapid single cell lysis was successfully achieved under a low-voltage alternating current [39]. Chemical cell lysis applies lysis buffer and induces high efficiency lysis to disrupt cells [40]. Several surfactants, such as sodium dodecyl sulfate (SDS) and Triton X-100, are introduced into a cell membrane to create pores within the membrane and lyse the cell. Jen et al. developed a microfluidic chip with arrays of microwells for single cell chemical lysis, and they found that cell membranes were gradually lysed as the lysis buffer containing 1% (v/v) Triton X-100 was injected [38]. Enzymatic cell lysis usually uses combination of enzymes to achieve complete dissociation of cells, which is the most mild method in reducing DNA breakage [41]. Proteinase, such as pepsin and trypsin, are applied to digest cytoplasm and histone-contained chromosomes. Quake’s group developed a microfluidic device and lysed single cells with pepsin at low pH to generate chromosome suspension. They neutralized them later with alkali on a pump-controllable platform for whole-genome molecular haplotyping [42]. Multiple factors, including cell type, downstream analysis, difficulty of genomic DNA (gDNA) purification, need to be taken into consideration for selection of the most appropriate lysis method [43].

### Nucleic acid amplification

The contents of nucleic acid, such as DNA or RNA, in individual cells is far below the quantity of sample needed for sequencing, thereby amplification process is essential for subsequent analysis. According to the previous report, there are approximately 10 pg of total RNA in a mammalian cell [44]. At present, there are two common amplification methods: whole-genome amplification (WGA) and whole-transcriptome amplification (WTA). These methods can significantly increase the quantity of total nucleic acids with high efficiency and low bias, generally from the nanogram to the microgram level [45]. The conspicuous difference is that WTA needs a reverse transcription process to produce cDNA samples. Approximately 10–20% of mRNA is reversely transcribed at this stage [46].

The WGA method currently available include degenerate oligonucleotide-primed polymerase chain reaction (DOP-PCR), multiple displacement amplification (MDA), and multiple annealing and looping-based amplification cycles (MALBAC) [47]. The currently available WTA methods include traditional PCR, modified PCR, T7-in vitro transcription (IVT) and Phi29 DNA polymerase-mediated RNA amplification [48].

In the single cell transcriptome analysis, cDNA amplification is never perfectly linear, resulting in a disproportional representation of all cDNAs in a cell. Thus unique molecular identifiers (UMIs) are now used to mark primary RNA to reduce amplification bias [49]. Moreover, the quality of amplified products is the key factor for development of single cell genomic and transcriptomic sequencing. Therefore, the quality of RNAs should be evaluated before sequencing, and the most frequently used method is Sanger sequencing [50]. Recent studies demonstrated that molecular analysis of DNA or RNA using droplet digital PCR (ddPCR) technique has advantages as compared to Sanger sequencing or real time PCR approaches [51, 52]. The third generation of polymerase chain reaction, ddPCR is a novel method for the absolute quantification of target nucleic acids, counts absolute DNA amounts by direct counting positive wells, providing better comparable results in different tests. The ddPCR independent of standard curve and cycle threshold (CT) value, has greatly improved the sensitivity, specificity and precision for the detection of trace nucleic acid and rare sequences [53].

### Single cell sequencing

Single cell sequencing is a new technology for high-throughput sequencing analysis of genetic material at individual cell level, which promotes rapid advances in revealing heterogeneity in cell subtype classification and physiology identification. Genome, transcriptome, and epigenome sequencing are fundamental constitutions that reflect single-cell developmental trajectory [54]. Single cell genome sequencing, that is scDNA-seq, reveals mutations and structural changes of the cell genomes, and highlights the somatic clonal structure and track the evolution and spread of the diseases [55]. Single cell transcriptome sequencing, namely scRNA-seq, can measure gene expression across the transcriptome at the single cell resolution, identifying biologically relevant differences in cell clusters [56]. Single cell epigenome sequencing focuses on the heritable changes in phenotype that do not change in the DNA sequence, which involves in DNA methylation, chromatin accessibility, histone modifications, and DNA folding, reflecting how genomic structure variation influences cellular phenotype [57]. Significantly, single cell bisulfite sequencing (scBS-seq) is served as a gold standard for investigation of DNA methylation. In addition, single cell assay for transposase accessible chromatin with high-throughput sequencing (scATAC-seq), and single cell multi-omics sequencing (scCOOL-seq) can analyze the chromatin state/nucleosome positioning and DNA methylation, which are successfully performed in microfluidic devices [58]. Although scDNA-seq is a powerful tool for complete quantitative sequencing with only a pair of DNAs in individual cells, there is only 1 or 2 DNA copy in a single cell. Therefore, the development of scRNA-seq has wide application as it can collect mRNA information in tissue samples and recognize where the transcript comes from [59].

Common steps required for the generation of scRNA-seq libraries involves reverse transcription into first-strand cDNA, synthesis of the second-strand cDNA, and cDNA amplification [60]. More RNA, cDNA and DNA sequence reads can be produced from genes that are highly expressed than weakly expressed genes in a sample. Therefore, RNA sequencing provides reads of gene expression, with the number of sequencing reads corresponding to the expression level of genes in a sample, which makes up a digital gene expression matrix for bioinformatic analysis. Each cell type possesses a unique transcriptome that can be demonstrated as a data matrix. Remarkably, current scRNA-seq enable defining the expression levels of all genes [61].

In the scRNA-seq method, all transcripts in individual cells are transformed into cDNA by reverse transcription. Some methods support the entire transcriptome sequencing, while others allow for sequencing the 5′ and 3′ ends of the transcriptome only. For instance, Smart-seq2 permits full read coverage of cDNA sequencing, and Fluidigm C1 microchamber-based system can automatically complete the Smart-seq steps, which can perform 96 parallel assays of single cell capture, lysis, reverse transcription, and preamplification [62]. Drop-seq, inDrop and Chromium system provide sequence information only for the 5′ or 3′ ends of the cDNA, and thus inapplicable in the analysis of alternative splicing patterns [63]. All of them aim to capture the original RNA molecules and then be amplified and sequenced uniformly and accurately [64].

### Data processing and analysis

A large amount of data will be obtained after high-throughput sequencing, and millions of reads or even more can be produced at one time. Therefore, efficient computer analysis for data processing is needed to satisfy with the requirements of subsequent analysis [65]. The data analysis process mainly includes quality control, mapping, standardization, cluster detection and subsequent analysis.

Quality control is a method to check the quality distribution of the entire reads and remove sequencing data that may be biased, ensuring that the sequencing results used for analysis are accurate and effective [66]. Mapping aims to map short sequences to reference sequences or genomes for recognition. The sequencing data may show zero result due to lost or transient gene expression. To eliminate the bias of cell specificity, data standardization is necessary after reads of sequence and mapping are completed [67]. Dimension reduction process is used to visualize the data in cluster detection, which distinguishes cells according to differences of gene expression profiles. Based on experimental design, subsequent analysis could be flexible and include gene ontology (GO), Kyoto encyclopedia of genes and genomes (KEGG), differential genes expression analysis and protein interaction network analysis [68].

## Principles of microfluidics for single cell analysis

**Fig. 3:**
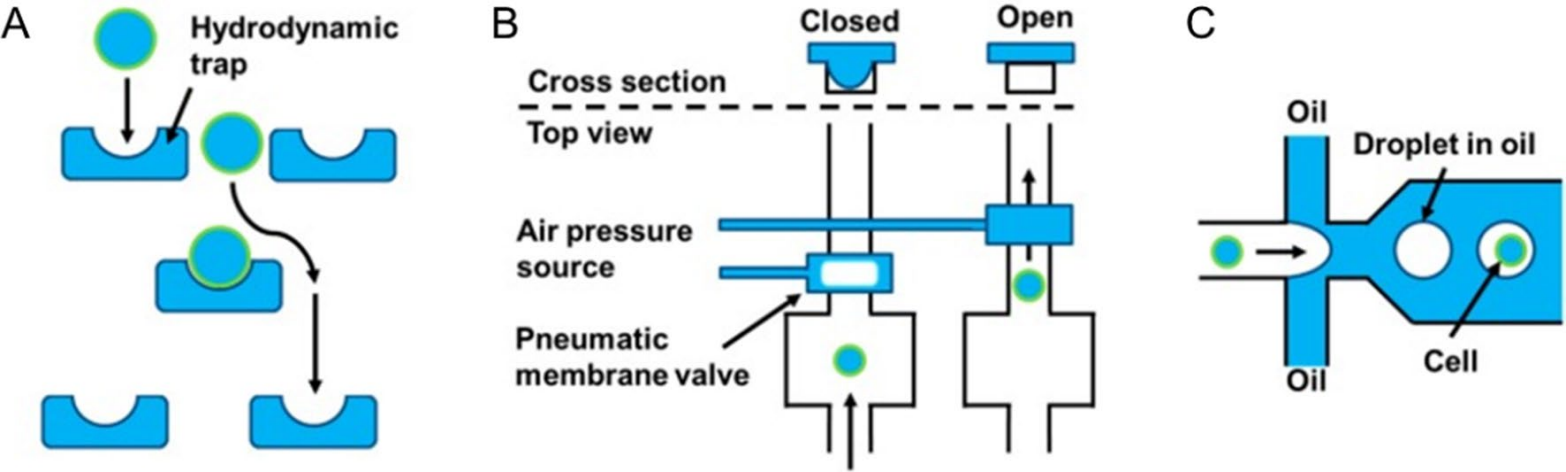
Different principles of microfluidics for single cell analysis. **A** traps-based method; **B** valves-based method; **C** droplet-based method. Adapted with permission from Gross A et al. [32]

Over the last decade, microfluidic system has made substantial contributions to dynamically monitoring cells and controlling cellular microenvironment [69]. Microfluidic chips can simulate the biological environment in vivo and allow for high-throughput analysis of cells. Besides, the flexible combination of multiple units enables the processes of cell injection, culture, capture, lysis and detection to be completed on a microfluidic chip [70]. Microfluidic chip can be broadly classified into three principles of technology: traps-based microfluidics, valves-based microfluidics, and droplet-based microfluidics (Fig. 3).

### Traps-based microfluidics

Microfluidic chips with cell traps have been designed and implemented for single cell studies. Traps-based microfluidic devices are capable of capturing single cell with high efficiency and culturing cell with low risk of pollution. Research showed that traps-based microfluidics possessed the highest efficiency compared with other cell isolation methods, with up to 97% of traps filled with a single cell [71].

Trapping of single cells by active or passive capture strategies at fixed positions have been widely used in microfluidic systems. Among various cell-trapping methods, the hydrodynamic traps have been tested and proved to be effective in capturing cells for culture purpose [72]. Hydrodynamic traps are passive structures in microfluidic channels, allowing only one cell to enter the trap. The size of the traps depends on the size of the cells under study. Double occupation is minimized through adjusting the trap size to the average cell size in a sample. Thus, this system can be operated using a large number of traps in parallel for high-throughput cell manipulation [73].

A hydrodynamic cell trap is a simple system where the cell is removed from a cell suspension stream after being stopped by the microscale structures, such as U-shaped structure. The cell traps consist of one or more U-shaped structures in a flow pool that capture single cells from a bulk cell suspension [74]. Di Carlo et al. presented a microfluidic-based dynamic single cell culture array that consisted of physical U-shaped hydrodynamic trapping structures to trap single cells. In their device, individual cells can be trapped and cultured under dynamic control of fluid perfusion [75]. Zhang et al. integrated the hydrodynamic U-shaped traps into a hand-held single cell pipet (hSCP) for rapid and efficient isolation of single cells from cell suspension without the need of micromanipulation under a microscope [76]. Cell-to-cell interaction plays a significant role in the progression of diseases and it is a remarkable property that can be analyzed using the hydrodynamic traps. Two traps were connected by a free space, allowing juxtacrine communication between cells. Frimat et al. presented a highly parallel microfluidic approach combining a differential fluidic resistance trapping method with a valving principle for homotypic and heterotypic single cell co-culturing [77].

### Valves-based microfluidics

Owing to their inherent advantages, such as fast speed, high efficiency, low reagent consumption and small instrument size, fully integrated microfluidic systems are a promising technology. In a fully integrated microfluidic system containing several operating steps, micro-valves are essential part for physical separation of each functional unit [78]. These valves are used to wall off particular regions of the channel network, allowing reaction chambers be generated and independent reactions be carried out. In addition, the valves can be opened and closed on demand, which allows complex manipulation. Valves-based microfluidic systems that allow automatic on-chip operation greatly help researchers realize a variety of experimental functions required for single cell analysis. Therefore, valves-based microfluidics significantly contributes to accurately simulate the dynamics of cellular microenvironment with high precision and control [79].

Microfluidic large scale integration (mLSI) enables the fabrication of microfluidic chips containing hundreds to thousands of pneumatic membrane valves that are similar to fluidic multiplexers array [80]. Valves-based microfluidic devices are designed by two separately cured polydimethylsiloxane (PDMS) layers with channels so that the pneumatic membrane valves are formed when the channels in the two layers are orthogonal to each other [81]. The flexibility of PDMS allows integration of membrane valves on complex microfluidic chips to create an intricate network of microchannels [82].

Micro-valves can be divided into four categories, including active mechanical, active non-mechanical, passive mechanical, and passive non-mechanical. Generally speaking, active mechanical micro-valves are most commonly used in microfluidic systems because of its best performance, while simple passive valves are more suitable in practical application [83]. Valves-based microfluidics was first developed by the Quake lab in 2000, leading to a breakthrough towards largescale microfluidic integration and automation [84]. Besides, they utilized mLSI technology for isolation of mRNA, synthesis of cDNA, and purification of DNA on a fully automated microfluidic chip [85]. The Maerkl lab implemented the highly multiplexed and automated microfluidic device for simultaneous kinetic characterization of 768 biomolecular interactions, allowing the purification and characterization of proteins [86]. Blazek et al. developed a mLSI platform for temporal and chemical control of cell cultures to study the rapid dynamics of protein phosphorylation [87]. Although valves-based microfluidics can overcome the drawback of limited operability, the device fabrication and complex operation increase the cost.

### Droplet-based microfluidics

With the rapid development of microfluidic chips, the technologies such as formation, splitting, merging, mixing, sorting and capture of microdroplets on chip are becoming increasingly mature [88]. It is worth noting that the microdroplet technology enables high-throughput screening with small sample amount, which can be applied to a variety of biological assays at the single cell level, including single cell cultivation, genomics and transcriptomics analysis, digital PCR, RNA-seq, antibody detection, drug delivery and screening, toxicity screening, and diagnostics [89–92].

On a microfluidic chip, droplets are generated with one liquid phase breaking off another immiscible liquid. Droplet-based microfluidics precisely manipulates the fluids in micro-channel and encapsulate individual cells with injection of carrier oil, so that droplets with well-controlled and uniform size can be generated rapidly and consistently [93]. Droplet-based microfluidics encapsulates individual cells and provides ideal microreactors, and each droplet is functionally equivalent to a well in a microplate but with a reaction volume a million times smaller [94]. Thousands of individual compartments can be generated per second, and surfactants are used to reduce surface tension and prevent fusion of droplets. Furthermore, droplets are intrinsically scalable for the reason that the number of microreactors is not limited by the physical dimensions of the chips [95].

**Fig. 4:**
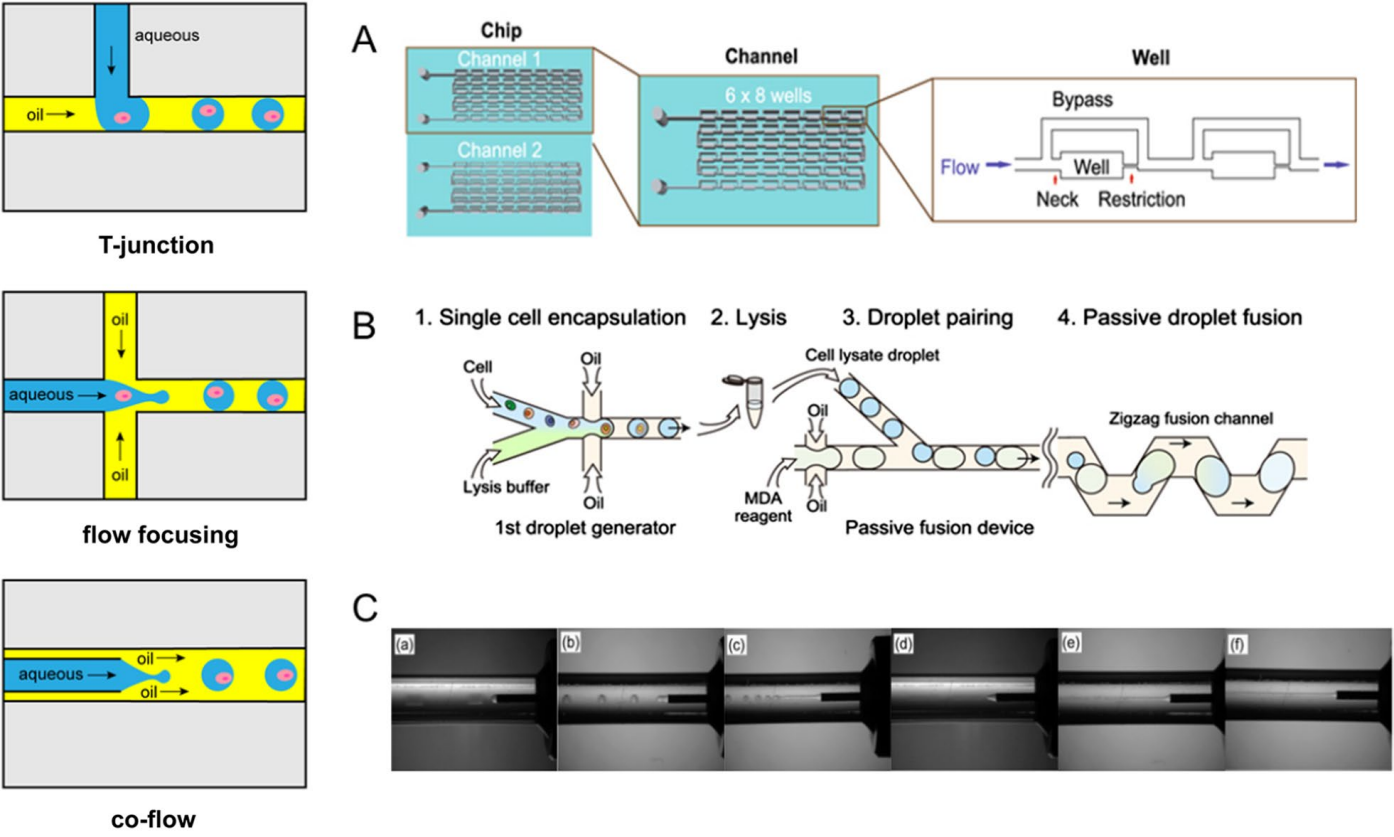
Common methods and applications for droplet generation, including T-junctions, flow focusing, and co-flow. **A** Droplets generated using T-junctions. The channel facilitated fluid flow in one direction, and droplets were formed in the well because of restricted flow, adapted with permission from Wong et al. [98]. **B** Droplets generated using flow focusing. In the droplet generator, single cells were mixed with lysis buffer and encapsulated into aqueous droplets in an oil-based emulsion, adapted with permission from Hosokawa et al. [99]. **C** Droplets generated using co-flow. Cell-enclosing droplets can be obtained from a high viscous aqueous solution under ambient co-flowing liquid, adapted with permission from Sakai et al. [100]

Droplets are generated from the use of two immiscible fluids, the carrier fluid and the dispersed fluid. Generation of droplets can be achieved with microfluidic devices using three different geometries, such as T-junctions, flow focusing, and co-flow (Fig. 4). The most common channel geometry of droplet generation is flow-focusing, where the injected dispersed phase (water phase) is sheared by the continuous phase (oil phase with surfactant) pumped from two side channels perpendicular to the water phase flow to form isolated compartments [96]. The droplet size can be toned by adjusting the flow rates of the above two phases of fluids. After droplet generation, different microfluidic modules can be used to manipulate droplets in a highly controllable manner, such as merge, split, re-loading, incubation, detection and sorting. Droplets can also be sorted according to the fluorescent signals to achieve phenotypic screening [97]. Based on this technology, highly monodisperse droplets can be generated continuously in large amounts, overcoming the limitations of low throughput in traditional single cell isolation methods.

A large number of literatures indicated that droplet-based microfluidics has developed as an excellent method for single cell culture. The common dispersed fluid for droplet generation can be culture medium with suspended cells, and droplets containing individual cells can be incubated in situ within the microfluidic devices and even transferred for off-chip cultivation [101]. Wu et al. exploited the principles of wetting behavior and capillarity to generate oil-covered droplet arrays with uniformly sized and regularly shaped droplets. Result shown that *Escherichia coli* could be encapsulated and cultured in droplets, and cell population and morphology could be dynamically tracked [102]. Hong et al. developed a droplet-based microfluidics to conduct drug screening in cells from cancer cell lines or primary tumors. In this way, single cells were dispersed in droplets and imaged within 24 h of drug treatment to assess cell viability (Fig. 4A) [98]. WGA is the first step for single-cell sequencing, but its poor throughput and accuracy impede development. Hosokawa et al. introduced single droplet multiple displacement amplification (sd-MDA) to achieve massively parallel amplification of single cell genomes, resulting in better quality of sequence data (Fig. 4B) [99]. Sakai et al. investigated the effects of various viscous aqueous polymer solutions on the diameter of the droplets generated in a co-flowing laminar stream (Fig. 4C) [100]. Nevertheless, droplet generation in a co-flow immiscible liquid for single cell sequencing has been rarely investigated.

Significantly, encapsulation of cells in droplets is random and relies heavily on Poisson statistics, thus droplet-based microfluidics technology may produce empty droplets and droplets containing multiple cells [103]. In order to produce high quality single cell droplet for studies, researchers should maximize the number of droplets containing a single cell by optimizing channel designs to improve the encapsulation rate of individual cells in generated droplets [104]. In addition, methods for millions of droplets stored off-chip in test tubes and analyzed or manipulated at later time points should be developed. The properties of the liquid–liquid interface surrounding the aqueous droplet compartment is seen as a major challenge, which is usually stabilized with a surfactant. Therefore, the droplets must be stabilized to prevent unnecessary merging of droplets. Different methods should be taken into consideration for the storage of droplets according with experimental requirements, whether in-line on chip or collected in vials and syringes requires a relatively stable emulsion [105].

## Comparative analysis of high-throughput scRNA-seq methods

It is reported that scRNA-seq has provided the characterization of transcriptional differences between coding RNA and non-coding RNA on a genome-wide level [106]. A number of scRNA-seq technological platforms are developed for high-throughput analysis of large numbers of cells.

### Microplate-based scRNA-seq technologies

Several scRNA-seq technologies rely on capturing or sorting individual cells into tubes or multi-well plates for single cell analysis. Specifically, disintegrated single cells were distributed to the microplates, where the single cells were cleaved, enriched with RNA, and finally collected together for sequencing [107]. Ziegenhain et al. generated data from 583 mouse embryonic stem cells to evaluate prominent scRNA-seq methods, and provided insights for technical platform selection and experimental scheme design [108]. Switching mechanism at the 5′ end of the RNA transcript (Smart-seq)/Smart-seq2 and Cell Expression by Linear Amplification and Sequencing (CEL-seq)/CEL-seq2 methods are briefly discussed below.

Smart-seq is developed for improving transcript read coverage and enhancing evaluation of single nucleotide polymorphisms. By contrast, Smart-seq2 can realize more read even full-length read coverage and higher sensitivity, which is known as the improved version of Smart-seq [109, 110]. A widely used commercial platform, Fluidigm C1 depends on the Smart-seq method, provides automated single cell lysis, RNA extraction, and cDNA synthesis for up to 800 cells in parallel on a single chip [6]. Smart-seq/Smart-seq2 can provide information regarding gene mutation, gene alternative splicing and allele-specific expression, which is widely used for single cell full-length mRNA analysis.

CEL-seq was the first to use linear amplification of cDNA transcription in vitro, which overcomes the limitation of small starting amounts of RNA through barcoding and pooling samples. Hashimshony et al. proved that CEL-Seq provided more reproducible, linear, and sensitive outcomes than PCR-based amplification methods [111]. Owing to adding a 5-base pair UMI upstream of the barcode, CEL-seq2 are more suitable for identifying PCR duplicates. The use of barcodes in CEL-seq enables 30% more genes detection and higher sensitivity, thus achieves better identification of single cells [112]. Unfortunately, these methods can only be utilized for the 3′ end sequencing, thus providing less transcriptomic information than full length transcript sequencing [113].

In addition to the above technologies, conventional scRNA-seq technologies include Single-cell Tagged Reverse Transcription Sequencing (STRT-seq), Massively Parallel RNA Single-Cell Sequencing (MARS-seq), Single Cell RNA Barcoding and Sequencing (SCRB-seq), allow for accurate, sensitive and importantly molecular counting of transcripts at single-cell level, aiming to minimize amplification bias and labeling errors and achieve high throughput process. Generally speaking, the equipment requirements and sequencing costs are minimal for microwell plate methods. SMART-seq provides full read coverage of cDNA sequencing and facilitates the analysis of alternative splicing patterns, identifying distinct transcripts with potentially different functions [114]. While other scRNA-seq methods provide sequence information only for the 3′ or 5′ ends of the cDNAs, which are inapplicable to the analysis of alternative splicing patterns [115]. These microwell plate-based scRNA-seq methods are labor-intensive and time-consuming when dealing with transcriptome quantification of large numbers of cells, thus microfluidic-based devices are needed for analyzing cells of different sizes.

### Microfluidics-based scRNA-seq methods

The introduction of the Fluidigm C1 microfluidics system in 2012 provides gene expression data for up to 96 cells in a single parallel run less than 24 h. While high-throughput Fluidigm integrated fluidic circuit (IFC) chip, introduced in 2015, enables examination of 800 cells simultaneously [116]. Use of microfluidics-based technologies enable hundreds of thousands of microdroplets to be generated at a lower cost. Surrounded by oil, these aqueous droplets have a volume equivalent to cell size and contain a bead with a cell-specific barcode and a single cell.

**Fig. 5:**
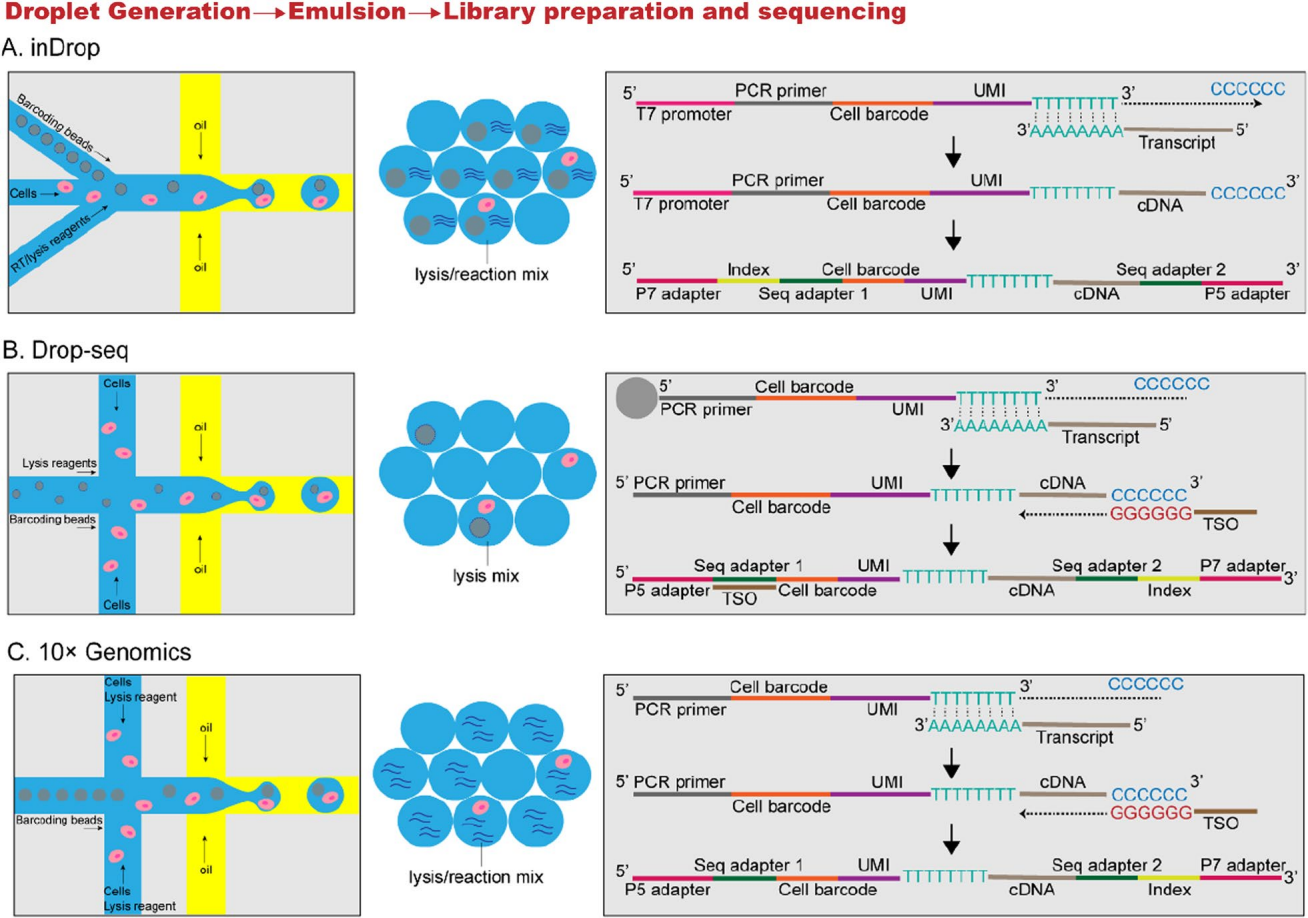
Comparison of droplet generation, emulsion, and library preparation and sequencing on the microfluidics-based scRNA-seq methods. **A** inDrop lyses single cells and then barcodes their mRNA with barcoded hydrogel microspheres in droplets; **B** Drop-seq applies barcoded beads capturing single cell mRNA and then released from the drops and performed reverse transcription; **C** 10× Genomics uses Gel bead in EMulsion (GEM) for encapsulating thousands of single cells, and then immediately lysis cells for reverse transcription

At present, the most popular high-throughput platform is droplet-based microfluidics, namely microdroplets. In this setting, individual cells are isolated into thousands of nanoliter droplets. Indexing droplets (inDrop) and Drop-seq were first put in application in 2015, both of them use the specific oil to generate droplets that contain lysis buffer, barcoded beads and cells. After cell lysis, the barcoded oligonucleotides hybridize to the poly(A) tails of the released mRNA, ensure the library preparation and sequencing [117]. For inDrop, the reverse transcription is performed within the drops, and then cDNAs are collected and amplified by IVT. For Drop-seq, the beads are released from the drops and pooled for reverse transcription, and then the cDNAs are amplified by PCR. These technologies allow the sequencing of thousands of cells in a cost-effective manner. Moreover, Nanowell technologies, including Gene expression cytometry (Cytoseq), microwell-seq, and Seq-well, were developed to enable massively parallel scRNA-seq, and show several benefits over droplet-based microfluidics including low reagent and sample volumes, and short cell-loading period. Split-pool ligation-based transcriptome sequencing (SPLiT-seq) allows efficient sample multiplexing, while scMT-seq simultaneously profiles both DNA methylome and transcriptome from the same cell. In this manuscript, we mainly discuss the inDrop, Drop-seq and 10× Genomics, which are summarized in Table 2, and comparison of droplet generation, emulsion, and library preparation and sequencing are showed in Fig. 5.

**Table 2:** Comparative analysis of microfluidic-based scRNA-seq methods

	inDrop [155]	Drop-seq [90]	10× Genomics [156]
Resemblances			
Isolation method	Droplet		
Number of cells	1000–10,000		
Cell barcode	Yes		
Unique molecular identifier (UMIs)	Yes		
cDNA coverage	3′ tag		
Differences			
Amplification method	In vitro transcription (IVT)	Template switching (PCR)	Template switching (PCR)
Cell barcode capacity	147,456 (384 × 384)	16,777,216 (4^12^)	734,000
Detection cost of 1000 cells	250 USD	100 USD	500 USD
Reaction in droplets	Cell lysis	Cell lysis	Cell lysis
	Primer release by UV	mRNA capture on beads	Primer release by bead dissolving
	mRNA capture		Reverse transcription
	Reverse transcription		Template switch
Reaction after demulsification	2nd strand synthesis	RT and template switch	PCR
	In vitro transcription	PCR	cDNA fragmentation and ligation
	RNA fragmentation	Tn5 tagmentation	
	PCR

#### inDrop technology

Reactions of inDrop are carried out in droplets, allowing the indexing of thousands of cells for scRNA-seq. inDrop technology introduces the oligonucleotides through hydrogel microspheres, and cell lysis and reverse transcription are carried out in droplets. The hydrogel beads dissolve in the droplets, releasing the oligonucleotides to hybridize the mRNAs, and the reverse transcription reactions are carried out within the drops. However, the major drawback of inDrop is the extremely low cell capture efficiency, which could detect only 20–50 copies of transcripts per cell [118]. This technology enables the scRNA-seq of large numbers of cells, which allows the identification of very rare cell types from heterogeneous populations. The library of Drop-seq contains 16 million barcodes, while inDrop technology produces only about 150,000 barcodes, meaning inDrop processes fewer cells per run. However, inDrop method is appropriate for analyzing very small tissue samples because it captures a higher percentage of cells than Drop-seq.

#### Drop-seq technology

Drop-seq has some common features with inDrop. Both of them allow a droplet encapsulating each single cell with a barcode. But Drop-seq is inclined to use barcoded beads, while inDrop utilizes barcoded hydrogel microspheres [119]. The oligonucleotide on beads consists of a handle sequence for amplification, a cell barcode that identifies all oligonucleotides from a single cell, a UMI, and an oligo(dT) sequence that captures single cell mRNA molecules. The beads, along with attached oligonucleotides and annealed mRNAs, are all released from the drops and combined into a single tube, and reverse transcription is then carried out [120]. Macosko et al. developed the Drop-seq technology where the transcriptomics of thousands of retinal cells were analyzed in nanoliter-sized aqueous droplets using barcoded microparticles [90]. Also, Weitz et al. used this method for subsequent analysis by NGS, which possessed low noise profile but only 7% mRNA capture efficiency.

#### Chromium system (10× genomics)

The Chromium system, namely 10× Genomics, was developed to enable digital counting of 3′ mRNA from thousands of single cells, which uses Gel bead in EMulsion (GEM) for encapsulating thousands of single cells. The Chromium system offers some advantages over the Drop-seq methods. First, the data obtained by this system are of higher quality than Drop-seq, because it detects more genes per cell and reduces technical noise. Second, the Chromium system provides more data of gene expression profile data for a much higher percentage of input cells. Third, the commercial Chromium system is easy for set-up and operation [121]. However, 10× Genomics has considerably high cost of reagents. Researches showed that only about 5% of Drop-seq input cells produce sequencing data, but over 50% of input cells into the Chromium system give rise to data, about a tenfold higher success rate than that of Drop-seq [59].

In summary, all three microfluidics-based scRNA-seq methods allow the bead-specific barcode incorporates into the cDNA, thereby enabling the subsequent DNA sequence reads to be aligned with a specific cell. Besides, all three methods allow the beads or cDNAs to be pooled together and processed through subsequent reactions, minimizing labor and reagent costs. In a cross-platform comparative study of Drop-seq, Fluidigm IFC and the Chromium system, Magella et al. found that all three platforms performed comparably, with each technology dividing mouse embryonic kidney cells into similar clusters with closely overlapping sets of biomarkers. The result showed that these technologies are considerably less expensive per cell than that in the Fluidigm methods [122]. Chromium System (10× Genomics) and Nadia (Dolomite Bio) are widely used as commercial platforms. Compared with 10× Genomics, both Drop-seq and inDrop are relatively low-cost and suitable for most high-throughput analysis, while 10× Genomics processes higher molecular sensitivity and precision with less technical noise.

## Microfluidics applications in single cell sequencing

Single cell gene expression profiling is rapidly becoming a standard analytical tool for researchers in various disciplines, such as cancer biology, neurobiology, immunology and cardiology. Quantification of cellular heterogeneity is important and has been used in numerous applications, such as analyzing the composition of solid tumors and understanding the development of embryos [123]. In this section, we discuss on the application of this technology for the study of cancer diagnosis and immune system diseases.

### Cancer diagnosis

A tumor contains a heterogeneous population of cells, including vascular cells, fibroblasts, invading immune cells and rapidly dividing cancer cells as well as cancer stem cells. More than 50% of the total DNA or RNA are extracted from the above cells [124]. The resulting gene expression profile of a pooled population of tumor cells therefore provides an ensemble average of the cell types present. Analysis of the pooled cell populations cannot identify specific cell types that express certain genes but instead provides an average gene expression profile of the multiple cellular components [125].

Single cell research allows the molecular differentiation of all cell types in the complex population combinations, and it is a potential way to better understand tumor heterogeneity [126]. With inherent advantages such as small sample volume, high sensitivity and fast processing time, microfluidics is well-positioned to serve as a promising platform for applications in oncology [127].

The gene expression patterns of stromal cells in the tumor microenvironment can provide prognostic value separate to that provided by the study of gene expression of intrinsic cancer cells. scRNA-seq technology is clearly a very useful tool to dissect the properties of the multiple cell types within and surrounding the tumor [128]. Sequencing of single cells is likely to improve several aspects of medicine, including the early detection of rare tumor cells, monitoring circulating tumor cells (CTCs), measuring intratumor heterogeneity, and guiding chemotherapy [129]. Thus, CTC isolation and sequencing, and individual tumor cell sequencing will be elaborated below.

#### CTCs isolation and sequencing

To study the intratumoral heterogeneity, rare cells, such as CTCs and cancer stem cells (CSCs), must first be isolated from cell culture or tissue samples [130]. Single cell analysis of CTCs provides an attractive surrogate biopsy of primary or metastatic tumors, as liquid biopsies can be collected in a minimally invasive procedure through a conventional blood sample [131]. At present, CellSearch^®^ remains the first choice to enrich CTCs for sequencing, which relied on epithelial cell surface biomarkers, such as epithelial cell adhesion molecule (EpCAM) or cytokeratins (CKs) [132]. But capture efficiency of this immunoaffinity-based CTC enrichment technology is not always stable. Lee group developed a novel microfluidic device, namely ClearCell^®^FX, which could enrich intact CTCs from the peripheral blood of cancer patients in a fully automated and high-throughput manner. ClearCell^®^FX utilized Dean Flow Fractionation (DFF) principle in a spiral microfluidics system to separate the larger CTCs from smaller blood cells [133].

**Fig. 6:**
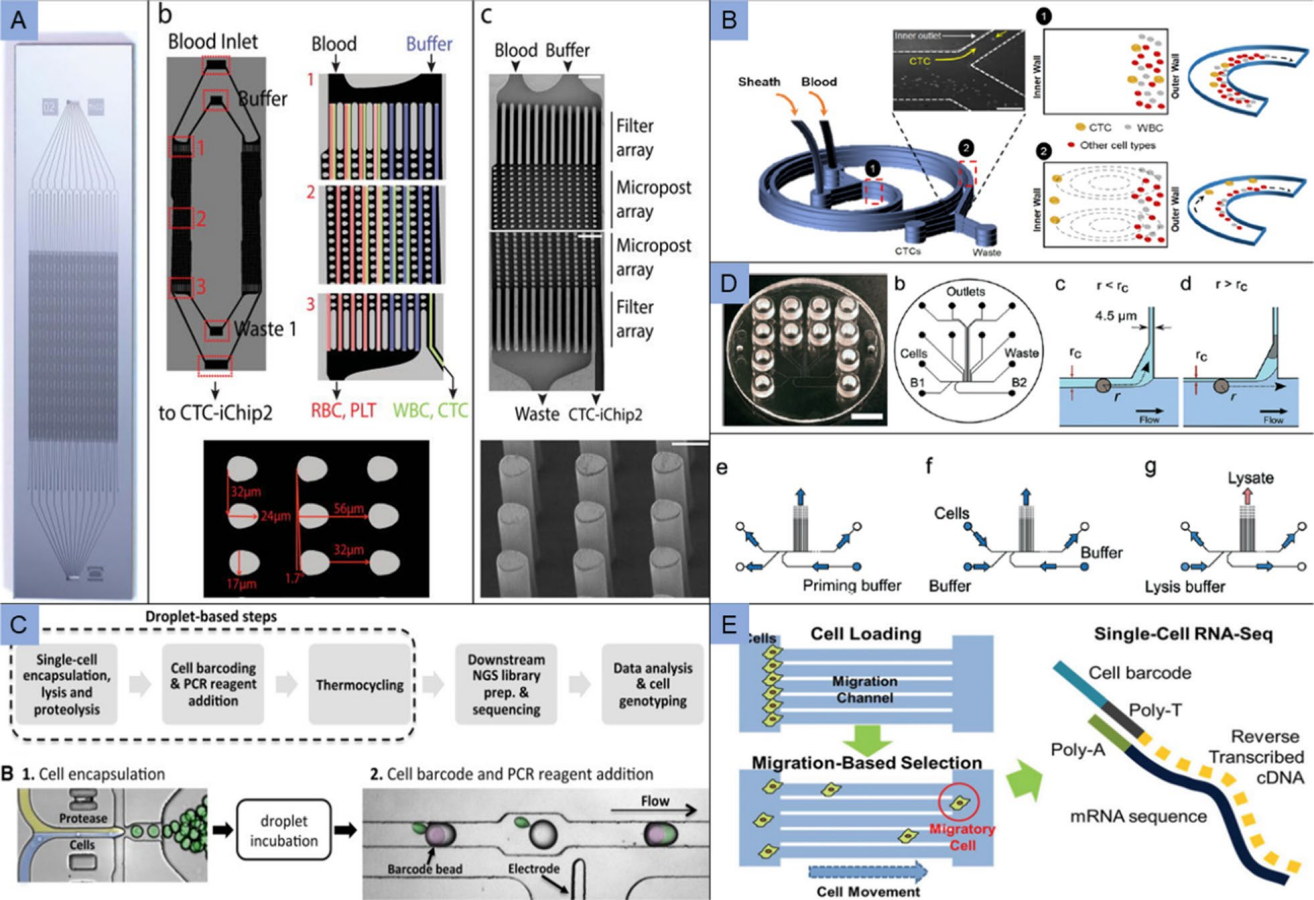
Application of microfluidic in cancer diagnosis. **A** The CTC-iChip composed of two separate and serial chips. Whole blood and buffer inlets enter from top corners, posts deflect nucleated cells away from smaller RBCs, platelets and plasma and toward the buffer. Adapted with permission from Karabacak et al. [134]; **B** Microfluidics for single cell sorting using DFF. The smaller RBCs and leukocytes exist the outer wall, while the larger CTCs focus along the microchannel inner wall. Adapted with permission from Vaidyanathan et al. [135]; **C** Protease-based droplet device. Cells are encapsulated with lysis buffer and incubated to promote proteolysis. The droplets containing the cell lysate are paired and merged with droplets containing PCR reagents and barcode-carrying hydrogel beads. Adapted with permission from Pellegrino et al. [141]. **D** Microfluidic device design and operation. The chip design is based on a hydrodynamic cell trap, and the trapped cell reduces the flow through the trap for the next incoming cell. Adapted with permission from Marie et al. [142]. **E** Microfluidic chip was performed to isolate migratory cells. Cells are initially positioned at the entrance of migration channels, and loaded cells migrated toward a gradient of serum chemoattractant in the center channel. Adapted with permission from Chen et al. [143]

The ability to isolate and analyze CTCs can importantly provide our understanding of cancer metastasis and treatment. Karabacak et al. isolated CTCs from blood samples by using tumor antigen independent microfluidic CTC-iChip, improving hematopoietic cell depletion. This modified CTC-iChip applied continuous deterministic lateral displacement (DLD) and inertial focusing for isolation of white blood cells and tumor cells, and then microfluidic magnetophoresis for immunomagnetic isolation of CTCs (Fig. 6A) [134]. Vaidyanathan et al. presented microfluidic approach using Dean flow fractionation (DFF) that combined of two microfluidic chips operating under inertial fluid forces and hydrodynamic focusing to rapidly isolate and selectively retrieve bulk and single CTCs from whole blood for downstream single cell analysis (Fig. 6B) [135].

However, several microfluidic technologies developed for CTCs enrichment based on either marker-dependent or cell size-dependent principles, which might ignore a large portion of small CTCs and have high WBC contaminations. Mao’s group developed integrated ferrohydrodynamic cell separation (iFCS) methods, based on contrast of cell magnetization in ferrofluids, for CTCs recovery with minimal WBC contamination. In this device, CTCs with almost zero magnetization were enriched and WBCs with high magnetization were depleted [136]. They also combined integrated inertial ferrohydrodynamic cell separation (i2FCS) method with single cell microfluidic migration assay to investigate the biological of invasive CTC phenotypes [137]. Mishra et al. developed ^LP^CTC-iChip platform based on magnetic sorting for removal of tagged hematopoietic cells and enrichment of viable CTCs. High magnetic gradients generated by ironfilled channels applied to inertial focusing flow of cells, which effectively deplete numbers of magnetically labeled leukocytes in microfluidic channels [138]. Novel microfluidic methods for high recovery and high throughput of CTCs enrichment are urgently needed. In addition, single cell sequencing of CTCs provides a mutational atlas for different cancers, thus offering the potential for clinical stratification of patients. Herein, several microfluidics technologies for CTCs isolation and single cell sequencing are summarized in Table 3.

**Table 3:** Microfluidics technologies for CTCs isolation and single cell sequencing

Sample type	Microfluidics device	Markers	Capture (%)	Sequencing technique	Key findings	Refs.
Breast cancer	Single-cell RNA sequencing (SCR-Chip)	EpCAM	93	SMART-Seq II	The sequencing data showed significant genetic differences between tumor cells and white blood cells. Tumor cells maintained a high consistency in the RNA panel, while there were large variations in WBC genes panel, which might be due to the presence of different subtypes in WBCs	[157]
Pancreatic cancer	CTC-iChip	CK CD45	95	ABI 5500XL	CTCs clustered separately from primary tumors and tumor-derived cell lines, showing enrichment for gene *Aldh1a2* and *Igfbp5*. Pancreatic CTCs exhibit a very high expression of stromal-derived extracellular matrix proteins, including *SPARC*	[158]
Lung cancer	Deterministic lateral displacement (DLD chip)	CK CD45	90	Illumina HiSeq	Six new somatic gene mutations in both single CTC and surgical specimen of this patient, namely *HIVEP2*, *SPATA21A*, *TUBGCP2*, *KCNG1*, *MIR4756*, and *ASMTL*	[159]
Breast cancer	ClearCell FX	CD45 CD31	80	Illumina MiSeq	Compared to peripheral blood mononuclear cell (PBMCs), CTCs showed elevated expression of breast cancer-specific markers *BRCA1* and *MDM2*, and a canonical epithelial cell marker *CDH1*	[160]
Prostate cancer	CTC-iChip	EpCAM CDH11 CD45	92	RNA-seq	A total of 711 genes were highly expressed in CTCs compared to primary tumors, with the most enriched being the molecular chaperone *HSP90AA1* and the non-coding RNA transcript *MALAT1*	[161]
Ovarian/colorectal/prostate/breast/pancreatic cancer	Sinusoidal microfluidics chip	EpCAM FAPα	80	Illumina HiSeq	*KRAS* mutational status in CTCs has been shown a high concordance with the primary tumor (~ 90%). *KRAS* mutations were detected in CTC^FAPα^ and CTC^EpCAM^ but not always in both CTC subpopulations	[162]
Lung cancer	Microfluidic chip with micropore arrayed filtration membrane	CK CD45	85	Illumina HiSeq X	Four common mutation sites were found between tissue and ctDNA samples before treatment, including CREBBP, ROS1, TP53 and EGFR. Moreover, oncogene HRAS mutated both in single CTC sample and ctDNA sample after treatment, rather than samples before treatment	[163]
Breast cancer	ClearCell FX	CK CD45	32.31	Single-cell whole-exome sequencing (WES)	There were a few hundreds of somatic mutations in the three CTCs, with only 16 overlapping mutations. Significantly mutated genes in pan-cancer BRCA1 and EPHA3 were found in CTC-1, and mutations in FGFR2 and ATM were found in CTC-3, indicating genomic heterogeneity among the CTCs	[164]
Prostate cancer	CTC-iChip	CD45 CD16 CD66b	93.8	Illumina NextSeq 500	No significant differences were evident between fresh and preserved blood for any of the 40 genes except for *KRT18*. Select genes in certain patients showed a trend toward increased expression	[165]
Prostate cancer	Celsee PREP100	CK CD45	79	Sanger sequencing	The p.K139fs*3 deletion of TP53 and p.T877A mutation of AR could be detected in the captured PC3 and LNCaP cells, respectively	[166]

#### Individual tumor cell sequencing

Intratumoral heterogeneity is a major obstacle in cancer treatment and a significant confounding factor in bulk-tumor profiling. Previous studies have demonstrated that cellular heterogeneity is closely associated with tumor metastasis, drug resistance, and clinical diagnosis [139]. For example, different responses of individual cells to drugs cause the emergence of drug-resistant cells, but only a small percentage (0.3%) of these cells have the ability to cause tumor recurrence [140]. The unambiguous identification of heterogeneous cell populations may be a major challenge, and the tumor evolution and acquisition of therapeutic resistance can be critically impacted by subclones.

Sequencing the genomes of individual cells enables the determination of genetic heterogeneity among cell populations. Pellegrino et al. developed a novel microfluidic droplet method, relying on cell-identifying molecular barcodes, to perform the longitudinal sequence of collected acute myeloid leukemia (AML) tumor populations (Fig. 6C). More than 16,000 individual cells were genotyped, showing a significant difference between bulk sequencing and actual subclones [141]. Marie et al. developed an injection-moulded valveless microfluidic device for trapping individual cells from colorectal cancer derived cell lines and colorectal tumors (Fig. 6D). After their single cell whole genomes were extracted in sub-nanoliter volumes, MDA was applied for preparing single cell DNA for pair-end Illumina sequencing, and they obtained genome coverages approaching 90% with almost correctly paired reads [142]. A microfluidic platform for cell migration was designed by Chen et al. to isolate migratory cells (Fig. 6E). The isolated cells were processed for scRNA-seq after functional validation. The result of Hydro-seq (microfluidic bead-cell pairing scheme) showed an elevated epithelial-mesenchymal transition (EMT) transcriptome profiles of migratory cancer cells [143]. Wang et al. performed the scRNA-seq analysis of U87MG and U87MG-EGFRvIII cells using 10× Genomics platform, and they found that the differentially expressed genes between groups were mainly enriched in DNA replication, DNA repair and angiogenesis [144]. In a word, detection of inherent heterogeneity at the single cell level has attracted wide publicity, which enables exploration for the properties of cancer and precise treatment.

### Immune system diseases

The immune system is a host defense system composed of various types of immune cells, which is responsible for protection of the organisms against diseases and maintain homeostasis [145]. Heterogeneity among immune cells is a big challenge for understanding the development of immune system. Moreover, heterogeneous cell populations could be caused by the recombination of B cells and T cells, hence the functional properties of immune system should be analyzed at the single cell level [146]. Recently, scRNA-seq has become a powerful tool for identifying new cell types and differentiation pathways of the immune system. For instance, using unbiased scRNA-seq of about 2400 cells in human peripheral blood, Villani et al. found new subtypes of dendritic cells and monocytes, and elucidated complex relationships among different cell types [147].

The microfluidic-based technologies with high spatiotemporal control and high-throughput made precision immunotherapy possible. Microfluidics allow the compartmentalization of individual cells and the amplification of specific genes or even the entire transcriptome. Therefore, microfluidic technologies contribute to understanding how the immune system gives rise to numerous potential responses against different pathogens [148]. Commercial microfluidics methods, such as Fluidigm C1, are shown to be extremely promising in exploring the molecular heterogeneity of the immune system.

**Fig. 7:**
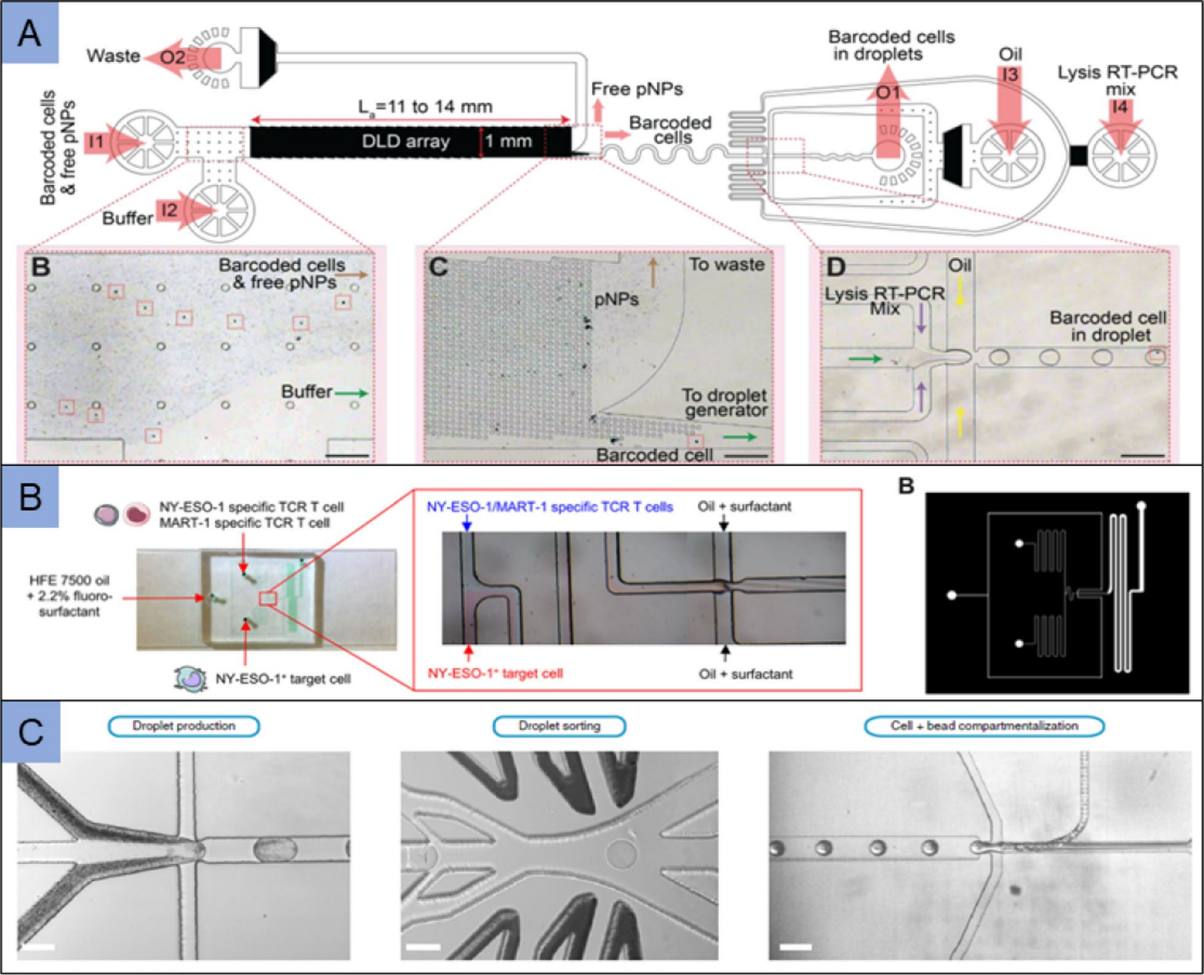
Application of microfluidic in immune system diseases. **A** These selectively labels peptide antigen-specific CD8 + T-cells are isolated in the microfluidic device integrated a DLD array for MATE-seq. Barcoded cells are encapsulated in water-in-oil droplets with lysis RT-PCR mixture, and collected for analysis. Adapted with permission from Alphonsus et al. [151]; **B** A flow-focusing droplet generator was used to generate aqueous in oil droplets containing co-encapsulated TCR T cells and target cells, which contributes to real-time monitoring of single TCR T cell activation after recognition of target tumor cells. Adapted with permission from Segaliny et al. [152]; **C** The droplet production, droplet sorting and co-compartmentalization of single cells with single beads in droplets are integrated in the CelliGO system, which is used for high-throughput single-cell activity-based screening and sequencing of antibodies. Adapted with permission from Annabelle et al. [154]

Adaptive immunity is based on peptide antigen recognition. T-cell receptor (TCR), a member of the immunoglobulin superfamily, recognizes the complex of processed antigens and major histocompatibility complexes (MHCs) [149]. It is crucial to isolate antigen-specific T cells from peripheral blood mononuclear cells (PBMCs) for TCR sequencing [150]. More and more studies demonstrated that microfluidic technologies can be utilized to analyze antigen-specific T cells at the single cell level. Alphonsus et al. reported microfluidic antigen-TCR engagement sequencing (MATE-seq), a single-stream Drop-seq derived method, applying for screening of barcoded pMHC-presenting NPs (pNPs) against CD8 + T cells. In this device, the pNP-labeled T cells were purified from free pNPs, and those cells were entrained into a droplet-generating microfluidic circuit as well as in-drop execution of RT-PCR (Fig. 7A). The MATE-seq method was used to capture and analyze rare, virus-antigen specific CD8 + T cells extracted from donor PBMCs [151]. Segaliny et al. developed a droplet microfluidics that co-encapsulating TCR T cells and target cells expressing the complex HLA/NY-ESO-1 antigen peptide in droplets. The droplet microfluidic device enables generating droplets with 120 μm in diameter, which containing co-encapsulated specific and non-specific TCR T cells together with target cells (Fig. 7B). After analysis, droplets containing activated T cells were sorted for downstream molecular analysis, including PCR and TCR sequencing. It is an effective means for quickly identifying candidate T cell therapeutics for future cancer treatments [152].

Antibodies are produced from the immune system in response to infection, and these antibodies can be widely used in diagnostic, therapeutic or research applications. Secreted by plasma cells, IgGs protect the body from infections by blocking molecular interactions and inducing complement/antibody-dependent cellular cytotoxicity [153]. Annabelle et al. presented CelliGO platform, a droplet microfluidic system, allowing phenotypic screening of IgG repertoires derived from large numbers of immune cells (Fig. 7C). CelliGO combines high-throughput screening with sequencing of paired antibody V genes, allowing recovery of approximately 450–900 IgG sequences from 2200 IgG-secreting activated human memory B cells [154].

Taken together, microfluidics-based technologies allow single immune cells for large-scale parallelized sequencing, which can not only achieve comprehensive analysis of the immune system, but also advance deeper understanding of biological processes and mechanisms.

## Conclusion

Single cell analysis is an extremely powerful tool for enhancing our understanding of inherent heterogeneity in during individual cells, facilitating the development of new diagnostic approaches, personalized medicines, and precise treatment of diseases. scRNA-seq provides detailed information of gene expression and could be used to discover and distinguish diverse cell types. Microfluidic technologies for single cell sequencing had been optimized with high sensitivity and high throughput. Although advances have been made in microfluidics-based single cell sequencing for diagnosis of cancers and immune system diseases, there are still rooms for improvement. For instance, the storage and test condition of samples in microfluidic device, the ratio of single cell encapsulation, detection instrument with faster and higher sensitivity, and more accurate data processing capability. Developing effective integrated microfluidic system for single cell manipulation and analysis will help personalized and precision medicine. New microfluidic technologies will be of great importance for research and development in biology and medicine fields.

## Data Availability

Not applicable.

## Abbreviations

NGS: Next generation sequencing
μTAS: Miniaturized total chemical analysis system
scRNA-seq: Single cell RNA sequencing
FACS: Fluorescence activated cell sorting
LCM: Laser capture microdissection
gDNA: Genomic DNA
WGA: Whole-genome amplification
WTA: Whole-transcriptome amplification
DOP-PCR: Degenerate oligonucleotide-primed polymerase chain reaction
MDA: Multiple displacement amplification
MALBAC: Multiple annealing and looping-based amplification cycles
IVT: T7-in vitro transcription
UMIs: Unique molecular identifiers
scDNA-seq: Single cell DNA sequencing
GO: Gene ontology
KEGG: Kyoto encyclopedia of genes and genomes
hSCP: Hand-held single cell pipet
mLSI: Microfluidic large scale integration
PDMS: Polydimethylsiloxane
sd-MDA: Single droplet multiple displacement amplification
Smart-seq: Switching mechanism at the 5′ end of the RNA transcript
CEL-seq: Cell expression by linear amplification and sequencing
IFC: Integrated fluidic circuit
inDrop: Indexing droplets
CTCs: Circulating tumor cells
CSCs: Cancer stem cells
EpCAM: Epithelial cell adhesion molecule
CKs: Cytokeratins
DFF: Dean flow fractionation
DLD: Deterministic lateral displacement
AML: Acute myeloid leukemia
EMT: Epithelial-mesenchymal transition
TCR: T-cell receptor
MHCs: Major histocompatibility complexes
PBMCs: Peripheral blood mononuclear cells
MATE-seq: Microfluidic antigen-TCR engagement sequencing
